# Adjuvant chemotherapy and survival in males aged 70 years or older with breast cancer: a population-based retrospective study

**DOI:** 10.1186/s12877-024-04861-1

**Published:** 2024-03-25

**Authors:** Yushuai Yu, Kaiyan Huang, Yushan Liu, Ruiliang Chen, Xin Yu, Chuangui Song

**Affiliations:** 1 Department of Breast Surgery, Clinical Oncology School of Fujian Medical University, Fujian Cancer Hospital, No.420, Fu Ma Road, Jinan District, 350014 Fuzhou, Fujian Province China; 2 Department of Breast and Thyroid Surgery, The Second Affiliated Hospital of Fujian Medical University, 362000 Quanzhou, Fujian Province China; 3 Fujian Medical University Union Hospital, 350001 Fuzhou, Fujian Province China

**Keywords:** Male breast cancer, Elderly patients, Chemotherapy, Clinical decision-making

## Abstract

**Background:**

Male breast cancer constitutes a minority of breast cancer diagnoses, yet its incidence has been on the rise in recent decades. However, elderly male breast cancer patients have been inadequately represented in clinical trials, posing challenges in treatment decisions. This study seeks to clarify the efficacy of chemotherapy in this demographic and identify the population most likely to benefit from such intervention.

**Methods:**

We conducted a retrospective analysis using the Surveillance, Epidemiology, and End Results (SEER) database, encompassing a total of 1900 male breast cancer patients aged 70 years or older. Among them, 1652 were categorized in the no-chemotherapy group, while 248 were in the chemotherapy group. A multifactorial logistic regression model was employed to investigate the determinants influencing the administration of chemotherapy in elderly male breast cancer patients. Additionally, the multivariate Cox proportional hazards regression model was applied to identify factors associated with outcomes, with overall survival (OS) as the primary endpoint.

**Results:**

Multivariate logistic regression analysis revealed that grade, tumor size, and nodal status were robust predictors for elderly male breast cancer patients receiving chemotherapy. Furthermore, the multivariate analysis demonstrated that chemotherapy conferred benefits compared to the no-chemotherapy group (HR = 0.822, 95% CI: 0.682–0.991, *p* = 0.040). Stratified analyses indicated that individuals with N+, poorly/undifferentiated grade, and stage II/III disease could derive benefits from chemotherapy. Upon further investigation of progesterone receptor (PR) positive patients, it was found that only stage III patients experienced significant benefits from chemotherapy (HR = 0.571, 95% CI: 0.372–0.875, *p* = 0.010). Conversely, in PR negative patients, both stage II (HR = 0.201, 95% CI: 0.051–0.792, *p* = 0.022) and stage III patients (HR = 0.242, 95% CI: 0.060–0.972, *p* = 0.046) derived benefits from chemotherapy.

**Conclusion:**

Adjuvant chemotherapy may benefit certain elderly male breast cancer patients, specifically those with positive lymph node status, poorly/undifferentiated grade, and PR-positive in stage III, as well as PR-negative expression in stage II/III. Given favorable physical tolerance, it is advisable not to hastily dismiss chemotherapy for these elderly male breast cancer patients.

**Supplementary Information:**

The online version contains supplementary material available at 10.1186/s12877-024-04861-1.

## Introduction

Male breast cancer constitutes a mere 1% of newly diagnosed breast cancer cases, signifying its rarity [1]. Over recent decades, there has been a gradual rise in the incidence of male breast cancer[2, 3]. In the context of male breast cancer, encompassing both in situ and invasive forms, it is noteworthy that an estimated 47–70% of the patients diagnosed are in the elderly age group [4–6]. Male breast cancer exhibits a higher genetic predisposition compared to female breast cancer, with a 10% susceptibility in men versus 5–7% in women [7, 8]. Common genetic mutations associated with male breast cancer include BRCA1, BRCA2, CHECK2, MLH1, MSH2, and MSH6, with BRCA2 being the most prevalent [9]. Men carrying a BRCA2 mutation face a lifetime risk of developing breast cancer of approximately 5–10% [7, 8]. Given the scarcity of clinical trial data pertaining to older male breast cancer patients, especially concerning the contentious use of chemotherapy, therapeutic options remain uncertain [10]. With an escalating life expectancy in the population, it is imperative to address the question of which individuals stand to gain from chemotherapy and how it influences male breast cancer outcomes.

A previous report, drawing from the SEER database, noted that elderly patients were less likely to undergo chemotherapy compared to their younger counterparts [10]. Considering the potential added toxicity of chemotherapy drugs in this specific age group, clinical decision-making often leans towards undertreatment, which may impact prognosis. Earlier studies have highlighted those elderly male breast cancer patients face a heightened risk of overall mortality in comparison to younger patients [11, 12]. Could this discrepancy be partially attributed to undertreatment? Additionally, it was observed that the majority of male breast cancer cases exhibit hormone receptor expression, with rare human epidermal growth factor receptor 2 (HER2) expression. Nearly 42% of tumors were categorized as luminal A, while 49% were classified as luminal B and HER2 negative [13]. Male breast cancer patients with hormone receptor-positive status are recommended to undergo adjuvant endocrine therapy [14, 15]. Despite the promising effects of endocrine therapy, is there still a necessity for applying chemotherapy in male breast cancer patients? Furthermore, two studies attempted to investigate the impact of chemotherapy on male breast cancer utilizing the SEER database and National Cancer Database [4, 12]. However, both studies conducted the analysis within the general population. Nevertheless, they did not fully resolve the clinical ambiguity. Neither of them compared the benefits of chemotherapy within subgroups other than stage, such as lymph node stage, different age groups, pathologic grade, and so forth. Moreover, a discrepancy exists between these two studies. While Hong Pan et al. concluded that progesterone receptor (PR) negative patients across all stages should receive chemotherapy, Siddhartha Yadav et al. found that only Estrogen Receptor (ER) positive patients in stage II-III can benefit from chemotherapy [4, 12]. Thus, who stands to benefit more from chemotherapy among elderly male breast cancer patients remain a critical question.

Management of older male patients with breast cancer not only depends on the disease itself, but is also complicated by comorbidities, drug tolerance, physical condition, and expected life expectancy [16–19]. Chemotherapy will be more significant as life expectancy continues to increase in recent years. To compensate for the lack of evidence, we used data from the SEER database to analyze the role of chemotherapy in elderly male breast cancer by different subgroup analysis according to stage, lymph node status, PR status, and histological grade. We believe that the results of this study will help make clinical decision-making and assist in scientific investigations.

## Methods

### Data source and study population

We used SEER*Stat version 8.3.8 to include patients. We included 1900 patients based on the following inclusion criteria: male; diagnosed between 1975 and 2017; diagnosed at the age of 70 or older; breast cancer as the sole primary malignant tumor diagnosis; American Joint Committee on Cancer (AJCC) seventh edition stages I-III. In this study, patients with distant metastasis or in situ disease were excluded. We categorized the patients into two groups: the chemotherapy group and the no-chemotherapy group based on whether chemotherapy was administered. Patient characteristics included race, marital status, laterality, histology, grade, AJCC stage, tumor size, nodal status, ER, and PR. In the study’s data source and population segment, we analyzed treatment modalities, specifically focusing on surgical operation methods and the application of radiation therapy.

### Outcome measurement

In our study, the primary outcome of interest was overall survival (OS), which was calculated from the date of diagnosis to the date of death, or censored at the last follow-up date. Censoring occurred for patients lost to follow-up or who survived until the end of the follow-up period. For patients still alive at the conclusion of our study, the follow-up duration was measured from the date of diagnosis to the study’s end. In cases of lost follow-up, the duration was computed from the date of diagnosis to the last recorded contact.

### Statistical analysis

We used the chi-square test to compare the differences in demographic and clinical characteristics between the chemotherapy group and the no-chemotherapy group. Collinearity analysis was conducted to assess the degree of multicollinearity among the independent variables [20, 21]. To quantify multicollinearity, the Variance Inflation Factor (VIF) was calculated for each predictor variable. The VIF measures how much the variance of an estimated regression coefficient increases if your predictors are correlated. VIF values exceeding 5 may warrant further investigation, as they indicate increasing multicollinearity. In instances where VIF values, specifically those exceeding 10, were observed, the approach adopted involved the removal of such variables from the model. Multifactorial logistic regression model was employed to explore the predictive factors for chemotherapy administration in elderly male breast cancer patients. We employed the log-rank test to ascertain whether there was a statistically significant difference in OS rates between patients who received chemotherapy and those who did not. We used the multivariate Cox proportional hazards regression model to calculate the hazard ratio (HR) with a 95% confidence interval (CI) to identify outcome-associated factors. Factors with a *p*-value greater than or equal to 0.05 in the univariate analysis were considered as candidate variables for the multivariate analysis. To further explore which elderly male breast cancer patients are in greater need of chemotherapy, we grouped them based on different tumor grades, AJCC stages, nodal status, as well as PR status. Statistical analyses were performed using R software version 4.3.1. All analyses were two-sided, and a *p*-value less than 0.05 was considered statistically significant.

## Results

### Baseline characteristics

In this study, 1900 patients were included, comprising 1652 in the no-chemotherapy group and 248 in the chemotherapy group (refer to Table 1). The median follow-up duration was 186 months (Interquartile Range: 164–208 months) for the no-chemotherapy group and 102 months (Interquartile Range: 81–123 months) for the chemotherapy group. Noteworthy differences were observed in AJCC stage distribution. Stage II cancers were more common in the chemotherapy group (43.1%) compared to the no-chemotherapy group (34.3%), while Stage I cancers were less frequent in the chemotherapy group (11.3%) than in the no-chemotherapy group (32.3%). Regarding tumor size (T stage), T1 tumors were more prevalent in the no-chemotherapy group (41.7%), whereas T2 tumors were more prominent in the chemotherapy group (47.2%). Nodal status demonstrated a notable difference, with N0 status being more common in the no-chemotherapy group (54.1%) compared to the chemotherapy group (27.4%). Conversely, N1 status was more prevalent in the chemotherapy group (33.1%) compared to the no-chemotherapy group (16.6%). The surgical approach also showed a significant difference, with mastectomy being more predominant in the chemotherapy group (72.6%) compared to the no-chemotherapy group (47.9%). Furthermore, radiation status revealed a notable difference, as a higher proportion of patients in the no-chemotherapy group did not receive radiation therapy (83.2%) compared to the chemotherapy group (60.5%).

**Table 1:** Baseline characteristics of patients with chemotherapy and no-chemotherapy

Characteristics		No-Chemotherapy (*n* = 1652)			Chemotherapy (*n* = 248)			Total (*n* = 1900)			P ^c^
		No	%		No	%		No	%		
Median follow-up (months) (Interquartile Range)		186 (164–208)			102 (81–123)			174 (153–195)			
Race	White	1436	86.9		206	83.1		1642	86.4		0.196
	Black	143	8.7		30	12.1		173	9.1		
	Other ^a^	73	4.4		12	4.8		85	4.5		
Marital status	Married	1113	67.4		183	73.8		1296	68.2		0.127
	Not married ^b^	469	28.4		56	22.6		525	27.6		
	Missing	70	4.2		9	3.6		79	4.2		
Laterality	Left	836	50.6		133	53.6		969	51.0		0.374
	Right	816	49.4		115	46.4		931	49.0		
Histology	Ductal	1275	77.2		207	83.5		1482	78.0		**0.026**
	Other ^d^	377	22.8		41	16.5		418	22.0		
Grade	Well	181	11.0		7	2.8		188	9.9		**< 0.001**
	Moderately	649	39.3		103	41.5		752	39.6		
	Poorly/undifferentiated	424	25.7		100	40.3		524	27.6		
	Missing	398	24.1		38	15.3		436	22.9		
Stage	I	533	32.3		28	11.3		561	29.5		**< 0.001**
	II	566	34.3		107	43.1		673	35.4		
	III	192	11.6		91	36.7		283	14.9		
	Missing	361	21.9		22	8.9		383	20.2		
Tumor size	T1	689	41.7		74	29.8		763	40.2		**< 0.001**
	T2	477	28.9		117	47.2		594	31.3		
	T3/4	120	7.3		32	12.9		152	8.0		
	Missing	366	22.2		25	10.1		391	20.6		
Nodal status	N0	893	54.1		68	27.4		961	50.6		**< 0.001**
	N1	274	16.6		82	33.1		356	18.7		
	N2/3	115	7.0		73	29.4		188	9.9		
	Missing	370	22.4		25	10.1		395	20.8		
Estrogen Receptor	Positive	1047	63.4		197	79.4		1244	65.5		**< 0.001**
	Negative	31	1.9		7	2.8		38	2.0		
	Missing	574	34.7		44	17.7		618	32.5		
Progesterone Receptor	Positive	964	58.4		175	70.6		1139	59.9		**< 0.001**
	Negative	100	6.1		25	10.1		125	6.6		
	Missing	588	35.6		48	19.4		636	33.5		
Surgery approach	Breast Conserving Surgery	119	7.2		6	2.4		125	6.6		**< 0.001**
	Mastectomy	791	47.9		180	72.6		971	51.1		
	Missing	742	44.9		62	25.0		804	42.3		
Radiation status	No	1374	83.2		150	60.5		1524	80.2		**< 0.001**
	Yes	278	16.8		98	39.5		376	19.8		

Note:

^a^ Other includes American Indian/Alaskan native and Asian/Pacific Islander and Unknown

^b^ Not married includes divorced, separated, single (never married), unmarried or domestic partner, and widowed

^c^ The *P* value of the Chi-square test was calculated between the chemotherapy and without chemotherapy groups, and bold type indicates significance

^d^ Other represents all pathological types other than invasive ductal breast cancer, including invasive lobular carcinoma, medullary carcinoma, mucinous carcinoma, intraductal papilloma, papillary carcinoma, tubular carcinoma, and so on

### Predictors of chemotherapy receipt

Collinearity analysis revealed that the variable ‘Stage’ exhibited high VIF values (10.59) in relation to the receipt of chemotherapy, indicating significant multicollinearity (refer to Supplement Fig. 1a). Consequently, ‘Stage’ was excluded from subsequent analyses. Following this exclusion, reassessment through collinearity analysis confirmed that all remaining variables demonstrated low VIF values, thus alleviating concerns of multicollinearity (refer to Supplement Fig. 1b).

**Fig. 1:**
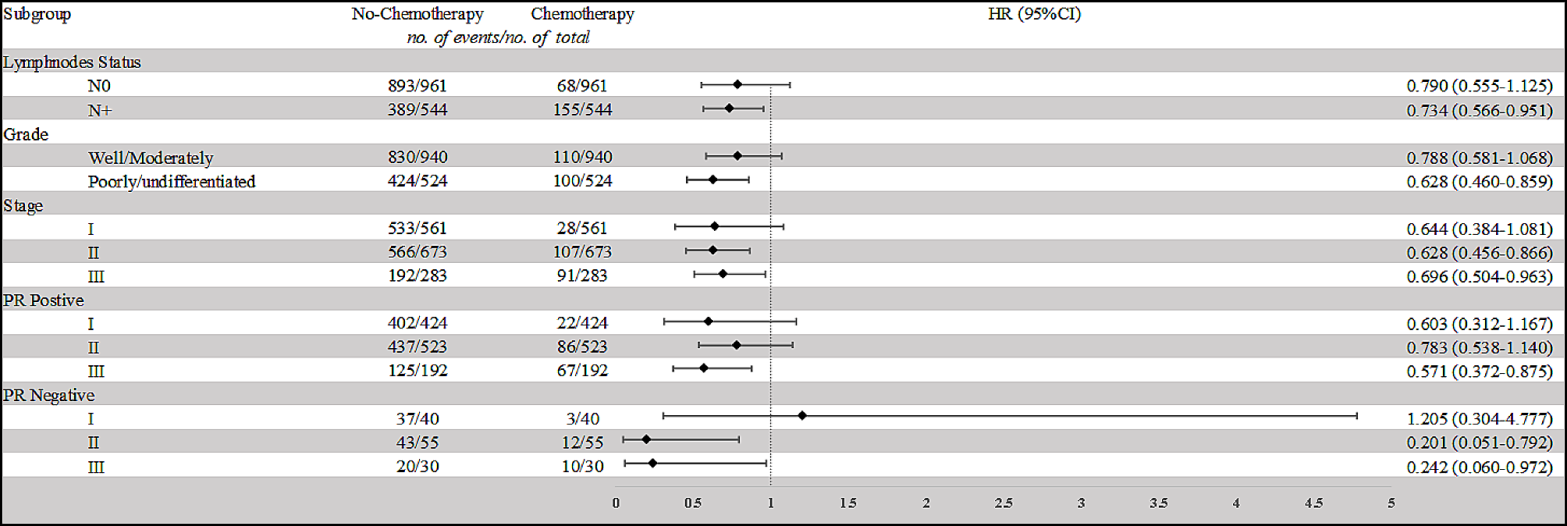
Chemotherapy effect on overall survival (OS) by subgroup Abbreviations: HR: hazard ratio

The multivariate logistic regression analysis identified several significant predictors for the receipt of chemotherapy in elderly male breast cancer patients (refer to Table 2). Grade, tumor size, and nodal status were also found to be significant predictors. Specifically, patients with moderately differentiated tumors had a higher likelihood of receiving chemotherapy compared to those with well-differentiated tumors (HR = 2.844, 95% CI: 1.262–6.409, *p* = 0.012). Patients with poorly/undifferentiated tumors had even higher likelihoods (HR = 3.773, 95% CI: 1.661–8.572, *p* = 0.002). Additionally, patients with positive nodal status (N1, N2/3) were more likely to receive chemotherapy compared to those with negative nodal status (N0) (HR = 2.889, 95% CI: 1.991–4.193, *p* < 0.001; HR = 6.158, 95% CI: 3.976–9.538, *p* < 0.001, respectively). Surgery approach and radiation status were also significant predictors. Patients who underwent mastectomy were more likely to receive chemotherapy compared to those who had breast-conserving surgery (HR = 2.947, 95% CI: 1.240–7.005, *p* = 0.014). Furthermore, patients who received radiation therapy were more likely to undergo chemotherapy (HR = 1.833, 95% CI: 1.313–2.558, *p* < 0.001).

**Table 2:** Predictors of receipt of chemotherapy using multivariate logistic regression analysis

Variables		Hazard Ratio (95% Confidence Interval)	P ^c^
Race	White	Reference	
	Black	1.401 (0.878–2.236)	0.157
	Other ^a^	1.164 (0.587–2.310)	0.663
Marital status	Married	Reference	
	Not married ^b^	0.635 (0.446–0.905)	**0.012**
	Missing	0.902 (0.417–1.952)	0.793
Histology	Ductal	Reference	
	Other ^d^	0.895 (0.602–1.332)	0.585
Grade	Well	Reference	
	Moderately	2.844 (1.262–6.409)	**0.012**
	Poorly/undifferentiated	3.773 (1.661–8.572)	**0.002**
	Missing	3.970 (1.581–9.969)	**0.003**
Tumor size	T1	Reference	
	T2	1.526 (1.077–2.162)	**0.017**
	T3/4	1.194 (0.697–2.045)	0.517
	Missing	0.720 (0.182–2.846)	0.640
Nodal status	N0	Reference	
	N1	2.889 (1.991–4.193)	**< 0.001**
	N2/3	6.158 (3.976–9.538)	**< 0.001**
	Missing	2.812 (0.723–10.939)	0.136
Estrogen Receptor	Positive	Reference	
	Negative	0.889 (0.325–2.433)	0.819
	Missing	0.412 (0.104–1.628)	0.206
Progesterone Receptor	Positive	Reference	
	Negative	1.428 (0.815–2.505)	0.213
	Missing	1.882 (0.518–6.835)	0.337
Surgery approach	Breast Conserving Surgery	Reference	
	Mastectomy	2.947 (1.240–7.005)	**0.014**
	Missing	1.162 (0.448–3.012)	0.758
Radiation status	No	Reference	
	Yes	1.833 (1.313–2.558)	**< 0.001**

Note:

^a^ Other includes American Indian/Alaskan native and Asian/Pacific Islander and Unknown

^b^ Not married includes divorced, separated, single (never married), unmarried or domestic partner, and widowed

^c^ The *P* value was calculated by multivariate logistic regression analysis and bold type indicates significance

^d^ Other represents all pathological types other than invasive ductal breast cancer, including invasive lobular carcinoma, medullary carcinoma, mucinous carcinoma, intraductal papilloma, papillary carcinoma, tubular carcinoma, and so on

### Comparison of survival between chemotherapy group and no-chemotherapy group

The multivariate Cox proportional hazard model was applied to assess the impact of various factors on OS in all patients (refer to Table 3). Marital status, Grade, Tumor size, Nodal status, Surgery approach, Radiation status, and Chemotherapy status exhibited a significant association with OS.

**Table 3:** Multivariate Cox proportional hazard model of overall survival in all patients. Note:

Variables		Overall Survival	
		Hazard Ratio (95% Confidence Interval)	P ^c^
Race	White	Reference	
	Black	0.946 (0.781–1.146)	0.570
	Other ^a^	0.818 (0.622–1.077)	0.152
Marital status	Married	Reference	
	Not married ^b^	1.456 (1.291–1.641)	**< 0.001**
	Missing	1.015 (0.761–1.354)	0.921
Histology	Ductal	Reference	
	Other ^d^	0.886 (0.778–1.009)	0.067
Grade	Well	Reference	
	Moderately	1.194 (0.963–1.479)	0.106
	Poorly/undifferentiated	1.427 (1.142–1.783)	**0.002**
	Missing	1.330 (1.046–1.693)	**0.020**
Tumor size	T1	Reference	
	T2	1.215 (1.057–1.395)	**0.006**
	T3/4	1.813 (1.448–2.270)	**< 0.001**
	Missing	0.608 (0.355–1.041)	0.070
Nodal status	N0	Reference	
	N1	1.197 (1.021–1.403)	**0.027**
	N2/3	1.612 (1.317–1.973)	**< 0.001**
	Missing	2.350 (1.387–3.981)	**0.001**
Estrogen Receptor	Positive	Reference	
	Negative	1.403 (0.927–2.123)	0.110
	Missing	1.223 (0.714–2.094)	0.464
Progesterone Receptor	Positive	Reference	
	Negative	1.193 (0.937–1.520)	0.152
	Missing	0.855 (0.507–1.441)	0.557
Surgery approach	Breast Conserving Surgery	Reference	
	Mastectomy	0.755 (0.583–0.979)	**0.034**
	Missing	0.814 (0.618–1.072)	0.144
Radiation status	No	Reference	
	Yes	0857 (0.736–0.997)	**0.046**
Chemotherapy status	No	Reference	
	Yes	0.822 (0.682–0.991)	**0.040**

^a^ Other includes American Indian/Alaskan native and Asian/Pacific Islander and Unknown

^b^ Not married includes divorced, separated, single (never married), unmarried or domestic partner, and widowed

^c^*P* value was adjusted by a multivariate Cox proportional hazard regression model and bold type indicates significance

^d^ Other represents all pathological types other than invasive ductal breast cancer, including invasive lobular carcinoma, medullary carcinoma, mucinous carcinoma, intraductal papilloma, papillary carcinoma, tubular carcinoma, and so on

In order to further clarify which population needs chemotherapy, we conducted subgroup analyses based on different nodal statuses, histological grades, staging, and PR statuses (refer to Table 4; Fig. 1). For patients with N0 status, the difference in OS between the chemotherapy and no-chemotherapy groups was not statistically significant (HR = 0.790, 95% CI: 0.555–1.125, *p* = 0.192). However, for patients with *N* + status, those receiving chemotherapy demonstrated a significantly improved OS compared to those without chemotherapy (HR = 0.734, 95% CI: 0.566–0.951, *p* = 0.019). Among patients with well/moderately differentiated tumors, there was no significant difference in OS between the chemotherapy and no-chemotherapy groups (HR = 0.788, 95% CI: 0.581–1.068, *p* = 0.124). Conversely, for patients with poorly/undifferentiated tumors, those receiving chemotherapy exhibited a substantially better OS compared to those not receiving chemotherapy (HR = 0.628, 95% CI: 0.460–0.859, *p* = 0.004). In Stage II, and Stage III cancers, patients who underwent chemotherapy demonstrated significantly improved OS compared to those who did not (*P* = 0.004, and *P* = 0.029, respectively); however, in Stage I patients, chemotherapy didn’t confer any benefit (*P* = 0.096).

**Table 4:** Comparison of overall survival between patients with chemotherapy and no-chemotherapy in specific tumor grades, stages, nodal status and progesterone receptor status using a multivariate Cox proportional hazard model

Variables	Overall Survival		
	Events	Hazard Ratio (95% Confidence Interval)	P ^a^
**N0 (** ***n*** ** = 961)**	613		
No-chemotherapy		Reference	
Chemotherapy		0.790 (0.555–1.125)	0.192
**N+ (** ***n*** ** = 544)**	373		
No-chemotherapy		Reference	
Chemotherapy		0.734 (0.566–0.951)	**0.019**
**Grade Well/Moderately ** ***n*** ** = 940)**	579		
No-chemotherapy		Reference	
Chemotherapy		0.788 (0.581–1.068)	0.124
**Grade Poorly/undifferentiated ** ***n*** ** = 524)**	382		
No-chemotherapy		Reference	
Chemotherapy		0.628 (0.460–0.859)	**0.004**
**Stage I ** ***n*** ** = 561)**	351		
No-chemotherapy		Reference	
Chemotherapy		0.644 (0.384–1.081)	0.096
**Stage II ** ***n*** ** = 673)**	429		
No-chemotherapy		Reference	
Chemotherapy		0.628 (0.456–0.866)	**0.004**
**Stage III ** ***n*** ** = 283)**	218		
No-chemotherapy		Reference	
Chemotherapy		0.696 (0.504–0.963)	**0.029**
**PR + Stage I ** ***n*** ** = 424)**	230		
No-chemotherapy		Reference	
Chemotherapy		0.603 (0.312–1.167)	0.133
**PR + Stage II ** ***n*** ** = 523)**	302		
No-chemotherapy		Reference	
Chemotherapy		0.783 (0.538–1.140)	0.202
**PR + Stage III ** ***n*** ** = 192)**	132		
No-chemotherapy		Reference	
Chemotherapy		0.571 (0.372–0.875)	**0.010**
**PR- Stage I ** ***n*** ** = 40)**	31		
No-chemotherapy		Reference	
Chemotherapy		1.205 (0.304–4.777)	0.791
**PR- Stage II ** ***n*** ** = 55)**	40		
No-chemotherapy		Reference	
Chemotherapy		0.201 (0.051–0.792)	**0.022**
**PR- Stage III ** ***n*** ** = 30)**	25		
No-chemotherapy		Reference	
Chemotherapy		0.242 (0.060–0.972)	**0.046**

^a^*P* value was adjusted by a multivariate Cox proportional hazard regression model and bold type indicates significance

Note: PR: Progesterone Receptor

To further analyze the effect of chemotherapy in stages patients with different PR statuses, we further segmented our population. The results revealed that among PR + patients, only those in stage III could benefit from chemotherapy (HR = 0.571, 95% CI: 0.372–0.875, *p* = 0.010). In contrast, PR- patients in both stage II and stage III showed a potential benefit from chemotherapy (PR- stage II: HR = 0.201, 95% CI: 0.051–0.792, *p* = 0.022; PR- stage III: HR = 0.242, 95% CI: 0.060–0.972, *p* = 0.046). Therefore, elderly male breast cancer patients who are PR + and in stage II-III, as well as PR- patients in stage I, may be exempt from chemotherapy.

## Discussion

The male breast cancer population presents a unique clinical challenge, characterized by a dearth of tailored clinical trial data and a propensity for treatment algorithms to confound clinicians. Moreover, advanced age is correlated with diminished survival prospects [11, 12]. This discrepancy is partially attributed to undertreatment, further exacerbating the issue. Presently, treatment approaches for elderly male breast cancer patients are predominantly extrapolated from guidelines established for elderly female breast cancer patients, encompassing a spectrum of interventions like surgery, endocrine therapy, radiotherapy, and chemotherapy [22, 23]. Among these modalities, chemotherapy engenders heightened controversy [24]. Our study, employing multivariable Cox regression, elucidates that not all elderly male breast cancer patients stand to benefit from chemotherapy. Thus, the judicious selection of candidates assumes paramount importance, mitigating the proclivity towards both overtreatment and undertreatment in clinical decision-making.

Our multivariable Cox regression analysis revealed a notable benefit of chemotherapy for stage II-III elderly male breast cancer patients. In a study investigating treatment patterns in stage I-III male breast cancer patients, Siddhartha et al. reported that the survival advantage associated with chemotherapy primarily manifested in patients with stage II-III disease. Although their findings were consistent with our own, it’s intriguing to contemplate whether all stage II patients, particularly in the context of elderly males, necessitate chemotherapy [12]. Past studies have underscored the prognostic significance of PR status in breast cancer patients [25]. This begs the question: how does PR status impact patients with negative versus positive expression within the same stage? To address this, we conducted a stratified analysis of stage II-III patients based on differing PR statuses. Our findings indicate that patients with PR-positive stage II may potentially forgo chemotherapy, as overall survival exhibited no significant improvement post-chemotherapy. Conversely, patients with PR-negative stage II-III stand to gain substantial benefits from chemotherapy. The conspicuous disparities between our conclusions and prior research may be attributed to older patients facing elevated risks of chemotherapy-related toxicity, mortality, reduced tolerability, and diminished chemotherapy sensitivity compared to their younger counterparts [4]. Patients with PR-negative breast cancer in stages II and III have better prognoses with chemotherapy, whereas PR-positive patients only show this benefit in stage III. This could be attributed to PR positivity being a favorable prognostic factor, while PR-negative breast cancers are more aggressive [26–28]. Previous biological experiments suggest that the absence of PR expression in tumors may indicate impaired growth factor signaling pathways, such as the phosphatidylinositol 3-kinase (PI3K)/Akt/mammalian target of rapamycin (mTOR) pathway, leading to increased invasiveness and resistance to therapy [29, 30].

Histological grade perennially constitutes a pivotal prognostic determinant in female breast cancer, wielding considerable influence over treatment decisions [31]. In the realm of male breast cancer, the role of histological grade remains relatively uncharted, with existing data yielding disparate conclusions [13, 32–34]. Our investigation reveals a noteworthy finding: within the poorly/undifferentiated grade cohort, the risk of death post-chemotherapy significantly diminishes compared to the well/moderately differentiated grade cohort. Given the heightened efficacy of cytotoxic chemotherapy in eradicating rapidly proliferating tumor cells, its administration remains imperative in the context of poorly/undifferentiated grade elderly male breast cancer. Notably, prior research suggests that roughly 33.5% of patients fall within the poorly/undifferentiated grade category, signifying a substantial portion of the population poised to derive meaningful benefits from chemotherapy.

Lymph node involvement constitutes the predominant adverse prognostic factor for male breast cancer patients. As demonstrated in prior studies, nearly half of elderly male breast cancer cases exhibit lymph node positivity. Within the broader population, numerous studies have underscored the substantial improvement in long-term prognosis conferred by chemotherapy for axillary lymph node-positive patients [23, 35]. Sharon H. Giordano et al.’s study on adjuvant systemic therapy in male breast cancer patients revealed a reduced risk of death in patients receiving adjuvant chemotherapy, with the greatest benefits observed in those with lymph node involvement; however, this finding did not attain statistical significance [23]. A prospective study with a 20-year follow-up similarly ascertained potential benefits of adjuvant chemotherapy in male breast cancer patients with positive nodes, though both studies lacked specific age range delineations [35]. Notably, our investigation delineates those elderly male breast cancer patients with lymphatic metastasis stand to gain substantial advantages from chemotherapy. Contingent on physical tolerance, it would be remiss for elderly male breast cancer patients, particularly those with lymph node positivity, to summarily forego consideration of chemotherapy.

To the best of our knowledge, this study represents the inaugural endeavor dedicated to discerning the impact of chemotherapy within this distinctive population. The findings, derived from an expansive patient cohort, furnish potential insights into the adjuvant chemotherapy prospects for elderly male breast cancer patients. Nevertheless, our study is not devoid of limitations. Firstly, the absence of HER-2 status in our analysis stems from restricted data availability. However, it is noteworthy that prior research indicates a majority of patients exhibiting HER-2 negativity, potentially mitigating bias in our conclusions. Secondly, constrained by the available information in the SEER database, we were unable to incorporate variables such as genetic predisposition mutations, specific chemotherapy regimens, dosages, anti-HER2 therapy, or endocrine therapy into our analysis. Given these limitations, future research, including additional data collection and clinical trials, will be essential to validate our findings.

## Conclusion

Adjuvant chemotherapy may benefit certain elderly male breast cancer patients, specifically those with positive lymph node status, poorly/undifferentiated grade, and PR-positive in stage III, as well as PR-negative expression in stage II/III. Given favorable physical tolerance, it is advisable not to hastily dismiss chemotherapy for these elderly male breast cancer patients.

### Electronic supplementary material

Below is the link to the electronic supplementary material.

Supplementary Material 1

## Data Availability

The dataset supporting the conclusions of this article is available in the Surveillance, Epidemiology, and End Results (SEER) database. The URL of the database is https://seer.cancer.gov/.
