# Supplementary material for: Adjuvant chemotherapy and survival in males aged 70 years or older with breast cancer: a population-based retrospective study
Source: BMC Geriatr. 2024 Mar 25;24:282. doi: 10.1186/s12877-024-04861-1 (PMC10964698; doi:10.1186/s12877-024-04861-1)
Supplement: Supplementary file 1 — Supplementary Material 1 [file 12877_2024_4861_MOESM1_ESM.doc]

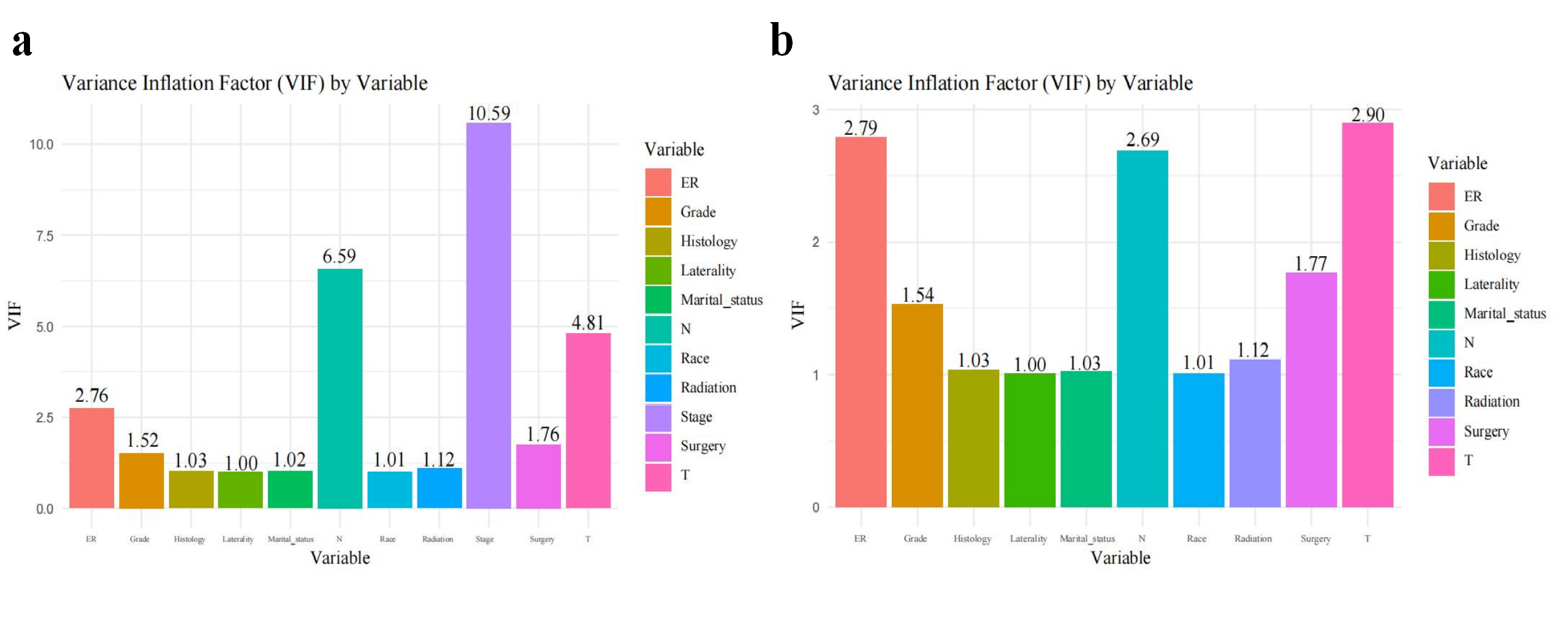


Supplement Figure 1: Collinearity analysis of different variables in relation to the receipt of chemotherapy; (a) With ‘Stage’ variable; (b) Without ‘Stage’ variable.
